# Impact of the COVID-19 pandemic on diagnosis and management of new brain tumours in children and young people (aged < 16 years old) in the UK

**DOI:** 10.1007/s00381-025-06928-9

**Published:** 2025-08-30

**Authors:** Sara Venturini, Kalsoom Akhter, Shivani Rajkumar, Midhun Mohan, Andrew Dapaah, Elizabeth Calton, Roland Casson, John-Paul Kilday, G. A. Amos Burke, Donald C. Macarthur, Ibrahim Jalloh

**Affiliations:** 1 Division of Neurosurgery, Department of Clinical Neurosciences, University of Cambridge, Cambridge, UK; 2 Cambridge University Hospitals NHS Foundation Trust, Cambridge, UK; 3 Nottingham University Hospitals NHS Trust, Nottingham, UK; 4 Children’s Brain Tumour Research Network (CBTRN), Royal Manchester Children’s Hospital, Manchester University NHS Foundation Trust, Oxford Road, Manchester, UK; 5 The Geoffrey Jefferson Brain Research Centre, University of Manchester, Manchester Academic Health Science Centre, Manchester, UK; 6 Cancer Research UK Clinical Trials Unit, University of Birmingham, Birmingham, UK

**Keywords:** Brain tumour, COVID-19, Paediatric

## Abstract

**Objective:**

This study aims to understand the impact of the COVID-19 pandemic on the diagnosis, management, and clinical outcomes of children and young people (CYP) with brain tumours in England.

**Design:**

UK-based retrospective observational study with two components. Nationwide brain tumour incidence data from the National Disease Registration Service was obtained (2015–2020) to study trends in newly diagnosed cases. Regional data from three paediatric neurosurgical centres was used to compare CYP newly diagnosed with a brain tumour before and those during the pandemic (March 2019–February 2021).

**Participants:**

No change in national incidence during the first year of the pandemic was seen. Regional data totalled 159 CYP, 88 diagnosed before the pandemic, 71 during (median age 7.0 years before, 7.8 years during the pandemic).

**Results:**

The commonest tumour types in both groups were low grade glioma (including pilocytic astrocytoma), medulloblastoma, and ependymoma. Symptoms at diagnosis were similar, the commonest overall being headache (52.3 vs 50.7%). Number of symptoms at diagnosis was comparable. Rates of complications (28.4 vs 40.8%, *p* = 0.129) and poor outcome (13.6 vs 22.5%; *p* = 0.143) were not significantly different. Time to diagnosis and treatment were similar between cohorts.

**Conclusion:**

This is the first UK study investigating the effects of the COVID-19 pandemic on CYP with a new brain tumour diagnosis. CYP diagnosed during the pandemic had comparable clinical presentations, times to diagnosis and treatment, and clinical outcomes. This suggests that acute paediatric neuro-oncology services were resilient in the face of pandemic-related restrictions and service adaptations.

**Supplementary Information:**

The online version contains supplementary material available at 10.1007/s00381-025-06928-9.

## Introduction

The COVID-19 pandemic rapidly became a worldwide health emergency in early 2020, being declared a global pandemic by the World Health Organization in March 2020 [1]. In paediatric oncology services, modifications became acutely necessary with a focus to minimise the risk of COVID-19 spreading within hospitals, while continuing to deliver acute services [2]. Travel restrictions imposed by lockdown measures represented a barrier to care access. These were combined with fear of infection, as hospitals were seen as a possible initiating sources [2].

While children and young people (CYP) globally represent a small proportion of deaths from COVID-19 [3], they have been indirectly impacted through disruption to everyday lives, such as via school closures [4]. Disruption to health services and perceptions had an impact. For example, Paediatric Emergency Department attendances decreased, with those attending doing so later than usual, with increased likelihood of requiring admission due to more advanced disease [5–7]. Data from an English paediatric neurosurgical centre showed that in early 2020, referrals to the neurosurgical inpatient team decreased when compared with the same period in previous years [8]. This is likely due to behavioural factors and may result in late presentation of treatable pathology, including brain tumours.

Childhood brain tumours represent one of the commonest childhood cancers and around 500 CYP are newly diagnosed annually in England [9]. More children die of brain tumours than any other solid cancers, and death may occur soon after diagnosis, from complications or recurrent/resistant disease [10]. Approximately 60% of long-term survivors have some neurological disability [11]. Delays in timely diagnosis can make treatment more complex, increasing the likelihood of death or disability [12, 13]. Late diagnosis can also have detrimental effects on subsequent relationships between families and their healthcare teams [12]. Few studies have investigated the effects of the pandemic on delays in diagnosis and treatment of paediatric solid tumours [2, 13]. An international study investigating the impact of the pandemic on paediatric cancer patients showed that children in low- and middle-income countries were primarily affected, compared to those in high-income countries [14].

Our study aims to understand the impact of the COVID-19 pandemic on diagnosis, management, and clinical outcomes of CYP with brain tumours in England. We hypothesised that disruption (actual and perceived) to normal health services had negative impacts on the patients’ journeys, including later presentation, compared to CYP utilising the same health services before the pandemic.

## Methods

### Nationwide data for CYP with new brain tumour diagnoses

Retrospective count data of CYP newly diagnosed with brain tumours in England were obtained from the National Disease Registration Service (NDRS, https://digital.nhs.uk/ndrs/), a database collecting data on cancers, rare diseases, congenital anomalies, managed by NHS Digital. Data on CYP (aged 0–14 years) with brain tumours was obtained from the National Cancer Registration Data, which combines several datasets (Hospital Episode Statistics, Death Certificates, Pathology datasets, Cancer Waiting Times, and Cancer Outcomes and Services). Data extracted was based on ICD-O-3 site codes: C70.0, C71.0-C71.9, C72.2-C72.5, C75.1-C75.3 and restricted to 0–14-year-olds to align with European age standardised rates. Data was available from January 2015 to December 2020.

### Regional data from selected centres

We collected regional data from hospital records at three paediatric neurosurgical/neuro-oncological centres in England (Cambridge, Manchester, and Nottingham) for all CYP newly diagnosed with brain tumours between 1 st March 2019 and 28th February 2021, aged between 0 and 16 years. Data were collected on patient demographics, tumour type, time from reported symptom onset to diagnosis, length of hospital stay, clinical outcomes including surgical complications, mortality, and discharge destination. Data were combined to form two patient cohorts: the group diagnosed before the start of the COVID-19 pandemic (1st March 2019 and 22nd March 2020) and the group diagnosed during the pandemic (23rd March 2020 to 28th February 2021).

### Statistical analysis

National count data and data from our three centres were summarised using descriptive statistics. Comparison between the two CYP cohorts diagnosed before and during the pandemic was made using the Mann–Whitney and Fisher’s Exact tests. Analysis was performed using RStudio (RStudio Team, 2020, package ‘tidyverse’). A *p*-value of < 0.05 indicated statistical significance.

Subgroup analysis was performed for pre-defined age groups: children below 5 years of age, children aged 5–11 years, and children aged 12 and over at diagnosis. These were based on the Brain Tumour Charity’s HeadSmart campaign, which highlights how presentation of CYP with brain tumour varies with age [15]. Subgroup analysis also investigated whether clinical outcomes varied between tumour types.

### Ethics

Ethical approval was granted by the NHS Health and Research Authority in December 2021 (IRAS: 295,305 HRA: 21/PR/1571).

### Patient and public involvement

Patients and the public were involved in the design of this research. Prior to the research study, we conducted a patient and public involvement co-design event with children and families. This event included presentations from the research team and a group discussion. Insights from this event informed the creation of our interview guide for a related qualitative study examining the pandemic’s impact on children and your people [16], and emphasised the need to understand why diagnostic delays occur for CYP and their families.

## Results

### National data on brain tumour diagnoses

**Fig. 1:**
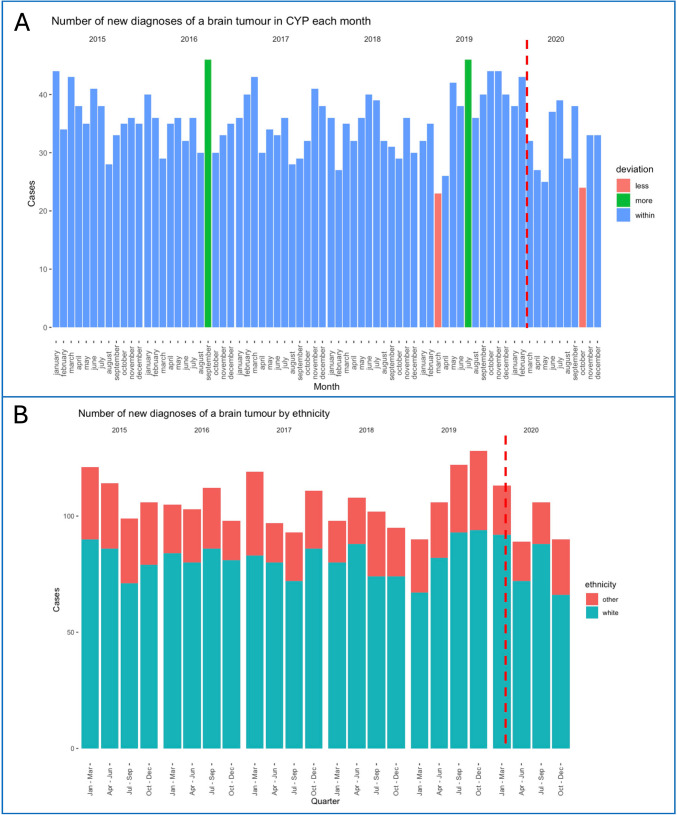
**a** Number of new brain tumour diagnoses in CYP each month in 2015–2020. Red: Months with number of new tumour diagnosis lower than two SD from the mean. Green: Months with number of new tumour diagnosis higher than two SD from the mean. **B** Number of new brain tumour diagnoses in CYP for each quarter in 2015–2020 by ethnicity, grouped as ‘white’ (red) and ‘non-white’ (blue). The dotted vertical red lines represent the start of the pandemic

In CYP aged 0–14 years, there were between 23 and 46 new brain tumour diagnoses per month during the 2015–2020 period, with a mean value of 35 (SD 5.35, SE 0.63). There were no obvious trends over time or in relation to the COVID pandemic. Cases fell below two standard deviations from the mean in March 2019 (23 cases) and October 2020 (24 cases) and rose above two standard deviations in September 2016 (46 cases) and July 2019 (46 cases). Ethnicity data showed no trends in the quarterly incidence of new diagnoses in white vs. non-white CYP between 2015 and 2020. Data is summarised in Fig. 1.

### Regional data from three neurosurgical sites

#### Baseline characteristics

A total of 159 CYP (80F; 79 M) with a median age of 7.1 years (0–16 years) were diagnosed with a new brain tumour during between 1 st March 2019 and 28th February 2021.

Eighty-eight CYP were diagnosed before and 71 during the pandemic. The two groups were similar with respect to age, gender, and range of histological diagnoses (Table 1). CYP diagnosed before the pandemic had a median age of 7.0 years (interquartile range [IQR] 4.85–12.3 years) and 7.8 years (IQR 3.2–12.8 years) during it. There was no statistically significant difference in age distributions at diagnosis between groups (Mann–Whitney, *p* = 0.986). The commonest tumour types in both groups were pilocytic astrocytoma, followed by medulloblastoma, followed by low grade glioma (not otherwise specified – NOS) and ependymoma in both time periods.

**Table 1:** Baseline characteristics of CYP diagnosed before and during the pandemic

	**Before**	**During**	**Significance (*p*-value)**
Total number diagnosed	88	71	-
Age at diagnosis (IQR)	7.0 (4.9–12.3)	7.8 (3.2–12.8)	0.986 (Mann–Whitney)
Sex (F/M)	46/42	34/37	0.855 (Fisher’s Exact)
**Type of tumours**			
Pilocytic astrocytoma	15	19	0.301 (Fisher’s Exact)
Medulloblastoma	15	9	
Low grade glioma (NOS)*	8	6	
Ependymoma	7	5	
Craniopharyngioma	6	4	
Ganglioglioma	4	1	
Diffuse midline glioma	4	3	
ATRT	3	3	
Anaplastic ganglioglioma	2	0	
Choroid plexus tumour	1	2	
DNET	2	2	
Germ cell tumour	1	3	
High grade glioma (NOS)*	1	1	
Other embryonal (NOS)*	1	1	
Pineoblastoma	0	2	
Other	15	9	

*NOS, not otherwise specified

#### Clinical presentation

The type and number of signs and symptoms at diagnosis were similar in the two cohorts (Table 2). The commonest symptoms were headaches (52.3% of those diagnosed before and 50.7% during the pandemic), nausea and/or vomiting (33.0% and 42.3%), followed by visual system abnormalities before the pandemic (27.3%) and motor abnormalities during it (28.2%). CYP diagnosed before the pandemic had a maximum of 3 symptoms/signs and those diagnosed during the pandemic had up to 5 symptoms at diagnosis. The number of symptoms was similar between the groups. In the pre-pandemic group, five patients had incidental diagnoses.

**Table 2:** Types and number of symptoms/signs at the time of diagnoses before and during the pandemic

Signs and symptoms	Diagnosis **before** the pandemic (%)	Diagnosis **during** the pandemic (%)	Fisher’s Exact *p*-value
Headache	46 (52.3%)	36 (50.7%)	0.593
Nausea and/or vomiting	29 (33.0%)	30 (42.3%)	0.289
Motor system abnormalities	19 (21.6%)	20 (28.2%)	0.436
Cranial nerve palsy (including ophthalmoplegia)	10 (11.4%)	8 (11.3%)	0.605
Visual system abnormalities	24 (27.3%)	15 (21.1%)	0.346
Seizures	9 (10.2%)	15 (21.1%)	0.060
Endocrine or growth abnormalities	3 (3.4%)	3 (4.2%)	0.706
Behavioural change (including lethargy)	7 (8.0%)	9 (12.7%)	0.420
Other	11 (12.5%)	12 (16.9%)	0.749
**Number of symptoms**			
0 (asymptomatic)	5 (5.7%)	0	0.245
1	31 (35.2%)	24 (33.8%)	
2	26 (29.5%)	24 (33.8%)	
3	21 (23.6%)	17 (23.9%)	
4	3 (3.4%)	5 (7.1%)	
5	0	1 (1.4%)	
missing	2.6%		

Both cohorts had similar symptom progression from onset to diagnosis (*p* = 0.402, Fisher’s Exact Test). Most patients had no increase in symptom numbers (67.0% before and 70.4% during the pandemic), and the remainder developed one additional symptom (25.0% vs 19.7%) or two (4.5% vs 9.9%)*.*

#### Time to diagnosis

**Fig. 2:**
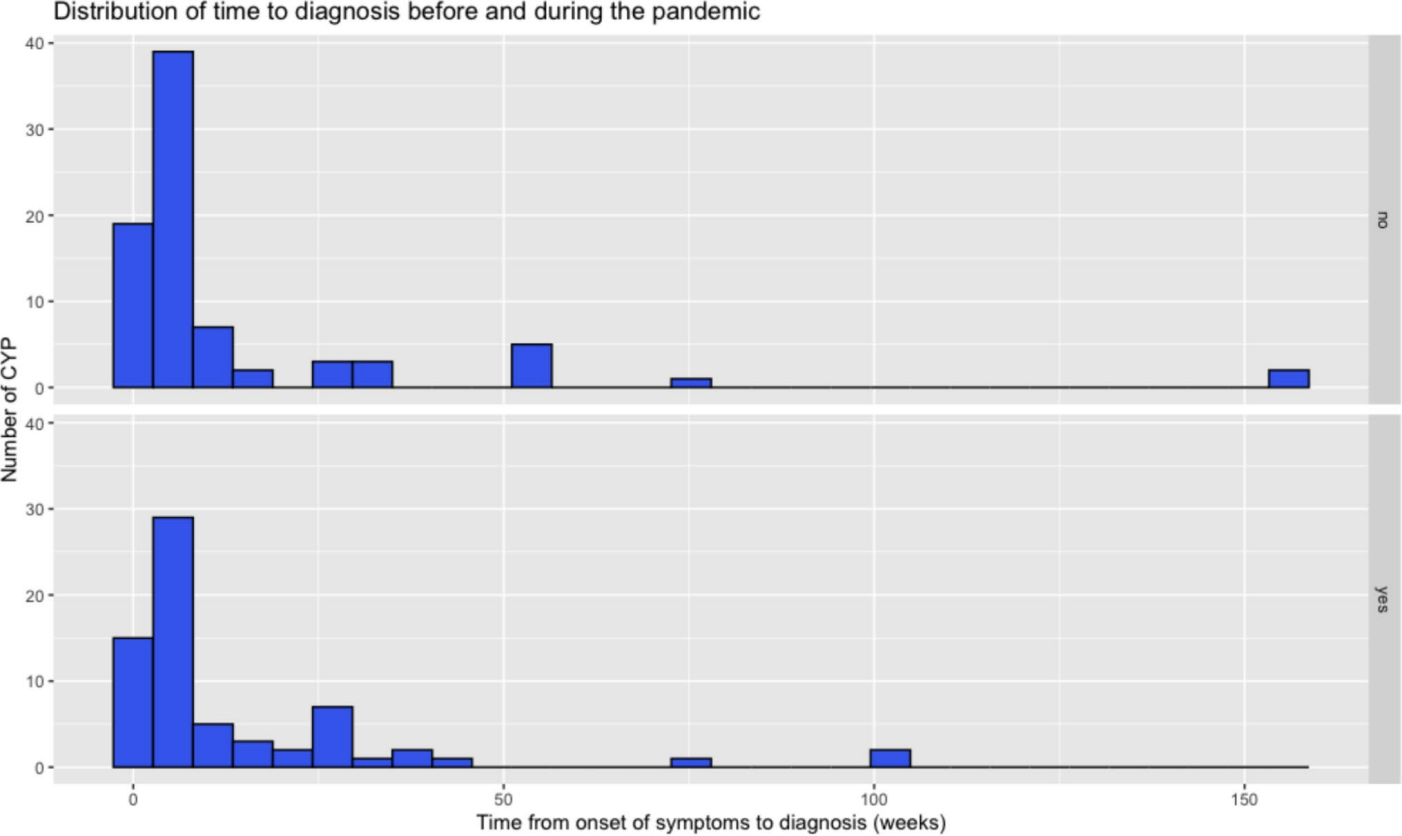
Distribution of the time to diagnoses (from onset of symptoms to diagnostic scan) in weeks. Above: time to diagnoses before the pandemic. Below: time to diagnoses during the pandemic

There was no statistically significant difference in the time to diagnosis, defined as the time from symptom onset to radiological diagnosis (Mann–Whitney, *p* = 0.758). The median time from symptom onset to diagnoses was 5 weeks (range 0–156) before the pandemic and 4 weeks (range 0–104) during the pandemic (Fig. 2).

#### Time to surgical and adjuvant treatment

Time to treatment, defined as the time from radiological diagnoses to primary surgical treatment for the brain tumour, was similar (Mann–Whitney, *p* = 0.642). The median time to treatment was 7 weeks (range 0.14–156) before and 5 weeks (range 0.15–105) during the pandemic.

Time to adjuvant treatment, defined as the time interval between primary tumour surgery and the start of adjuvant treatment (chemotherapy/radiotherapy), was also comparable (Mann–Whitney, *p* = 0.571). The median time to adjuvant treatment was 35 days (range 3–90 days) before and 28.5 days (range 5–150 days) during the pandemic.

#### Complications and clinical outcomes

Rates of urgent CSF diversion requirement before or at primary tumour surgery were similar between cohorts (22.7% before and 19.7% during the pandemic, Fisher’s Exact *p* = 0.700). The distribution of CSF diversion type used (external ventricular drain, endoscopic third ventriculostomy, or shunt insertion) did not vary significantly (Fisher’s Exact, *p* = 0.095). Values were 7 vs 11 for EVD, 11 vs 3 for ETV, and 1 vs 0 for shunt insertion before/during the pandemic.

Overall, complication rates following primary brain tumour surgery were similar when comparing CYP diagnosed before (25, 28.4%) with those diagnosed during the pandemic (29, 40.8%), Fisher’s Exact *p* = 0.129 (Table 3). Neurological complications and complications relating to cerebrospinal fluid (CSF), namely CSF leaks and post-operative hydrocephalus requiring treatment (shunt surgery), showed a trend towards higher numbers during the pandemic but this was not statistically significant. Rates of return to theatre within 30 days of primary surgery were comparable.

**Table 3:** Complications and clinical outcomes before and during the pandemic

Complications and clinical outcomes	Diagnosis **before** the pandemic (%)	Diagnosis **during** the pandemic (%)	Fisher’s Exact *p*-value
Requirement for urgent CSF diversion	20 (22.7%)	14 (19.7%)	0.700
Complications (any)	25 (28.4%)	29 (40.8%)	0.129
**Specific complications**			
Neurological	11 (12.5%)	14 (19.7%)	0.274
Wound or infection	5 (5.7%)	4 (5.6%)	1.000
CSF	10 (11.4%)	14 (19.7%)	0.182
Other	4 (4.5%)	3 (4.2%)	1.000
Return to theatre within 30 days for second examination	3 (3.4%)	6 (8.4%)	0.190
Return to theatre within 30 days for complication	8 (9.1%)	10 (14.1%)	0.451
**Clinical outcomes**			
Poor outcome (died or new deficit)	12 (13.6%)	16 (22.5%)	0.143

Clinical outcomes at discharge were similar when looking at the distribution of four clinical outcomes groups: (1) CYP who were well (a well-child without residual symptoms, *n* = 62, 70.5% before and *n* = 42, 59.2% during the pandemic), (2) CYP with a residual deficit (a child with residual deficit which had been present pre-operatively, *n* = 14, 15.9% before and *n* = 13, 18.3% during the pandemic), (3) CYP with a new deficit (new deficit post-operatively, *n* = 11, 12.5% before and *n* = 13, 18.3% during the pandemic), and (4) CYP who died (*n* = 1, 1.1% before and *n* = 3, 4.2% during the pandemic), Fisher’s Exact, *p* = 0.613. When comparing good outcomes at discharge (well or residual deficit) with poor outcome (new deficit or death), the group diagnosed during the pandemic showed a possible non-significant trend towards worse outcomes (Fisher’s Exact, *p* = 0.213).

#### Length of stay and discharge destination

Length of hospital stay was similar between cohorts. Median length of stay was 9.5 days (IQR 4–19.2) before and 8 days (IQR 4–15.8) during the pandemic (Mann–Whitney, *p* = 0.706). Most children were discharged home after the index hospital admission (Fisher’s Exact, *p* = 0.148, *n* = 84, 95.5% before and *n* = 67, 94.4% during the pandemic). Few patients were transferred to other hospitals (*n* = 3 before, *n* = 1 during the pandemic).

#### Subgroup analysis

Detailed results for the subgroup analyses are found in *Supplementary Materials*.

##### Age groups

Age groups broadly represent pre-school children (below 5 years), school-aged children (aged 5–11 years), and adolescents (12 years and above). There were no statistically significant differences in number of symptoms at presentation before/during the pandemic in age groups of less than 5 years and 5–11 years (Fisher’s Exact, *p*-values all > 0.05). In the age group 12 years and older, children diagnosed during the pandemic had a higher rate of nausea and/or vomiting (Fisher’s Exact, *p* = 0.002) and motor system abnormalities (Fisher’s Exact, *p* = 0.029) at diagnosis.

Rates of urgent CSF diversion, complications, and clinical outcomes were similar in all three age groups (Fisher’s Exact, *p*-values all > 0.05).

##### Tumour types

Subgroup analysis was performed for the three most common tumour types (pilocytic astrocytoma, medulloblastoma, and ependymoma). There was no statistically significant difference between rates of complication, return to theatre, and discharge outcome for any of the three tumour types (Fisher’s Exact, *p*-values all > 0.05 for pilocytic astrocytoma, ependymoma, medulloblastoma).

## Discussion

This study aimed to understand the impact of the COVID-19 pandemic on the diagnosis, management, and clinical outcomes of CYP with brain tumours in England, utilising nationwide incidence data from the NDRS and granular data from consecutive patient diagnosed at three paediatric neurosurgical referral centres. Combining the two datasets provides broad national-level trends on new diagnoses from the NDRS database, framing the context for the more detailed analysis coming from selected paediatric neuro-oncology centres, where custom data collection allowed investigation of specific outcomes.

Monthly incidence rates for England remained overall stable during the first year of the COVID-19 pandemic compared to previous years [9]. Possible downward fluctuations seen at times of lockdown were not significant. There was no significant increase or decrease in the number of new diagnoses for either ethnic group. Ethnicity was investigated as existing studies suggest that ethnic minorities were worse impacted by COVID-19 in terms of disease severity and access to services [17, 18]. However, it is important to recognise that other related factors such as socioeconomic status and geographical proximity may also influence access. A study based in the USA showed a decrease in incidence rates for brain tumours during the first months of the pandemic [19]. This decrease, seen in children younger than 14 years, was larger in ethnic minorities (40–45% decrease in April 2020 compared to April 2019). Authors suggested that these apparent decreases resulted from disruption to medical care and restrictions implemented to mitigate the spread of COVID-19 [19].

Studies from Turkey and Italy demonstrated that COVID-19 restrictions led to diagnostic and treatment delays for paediatric solid tumours [2, 13, 20]. Similarly, a US-based case series showed that paediatric solid tumours diagnoses decreased during the COVID-19 pandemic, and patients were admitted with more advanced disease [21]. Our data from England did not show a decrease in brain tumour numbers during the first pandemic year. This might be explained by the severity and type of symptoms, as parents are less likely to delay seeking medical care even during the pandemic [19]. Additionally, health system structure and referral pathways must be considered; the National Health Service is fully public, differing from other health systems where the referenced studies took place. It is possible that referral pathways were differently affected in different health systems.

Data was collected at three paediatric neuro-oncological centres for 2 years: the year just before the start of the COVID-19 pandemic (March 2019–March 2020) and the first year of the pandemic (March 2020–February 2021). Total numbers of new diagnoses in CYP were higher in the year before the pandemic than during it (88 vs 71). The most common tumours seen were pilocytic astrocytoma, medulloblastoma, other low grade glioma, and ependymoma, in line with published epidemiological data [22].

CYP diagnosed in both time periods had similar number of symptoms at both onset and hospital presentation. Similarly, time to diagnosis remained constant at a median 5 weeks before and 4 weeks during the pandemic. This finding contrasts results from previous studies showing delays in childhood cancer diagnosis during the pandemic [6, 13, 23]. However, our study corroborate those of a study from Canada, performed in a similar publicly funded health system, where no changes in childhood cancer incidence, time to diagnosis and severity at diagnosis, and early mortality rates during the first year of the pandemic [24]. The median time to diagnosis was in line with that reported in pervious studies [25, 26].

Headache was the most common symptom overall, followed in order by nausea and/or vomiting, visual system abnormalities, and motor system abnormalities. This distribution was similar to that reported in previous studies [11, 27]. Brain tumours can present with insidious symptoms, especially in young children who are unable to describe these. We performed subgroup analysis to explore whether the pandemic impacted children of different ages differently, for example due to school closures. We found that CYP aged 12 years and above diagnosed during the pandemic were more likely to have nausea and/or vomiting and motor system abnormalities compared to those diagnosed before the pandemic.

Time to treatment and time to adjuvant treatment following primary tumour surgery were not prolonged during the pandemic. This differs from results of a global survey describing changes to paediatric cancer care delivery including reduced surgical capacity and interruptions or unavailability of radiotherapy and chemotherapy treatments [28]. Multiple factors could contribute to this, including the centralised nature of neurosurgical and neuro-oncological treatments in England, and the CCLG (Children’s Cancer and Leukaemia Group) proactive establishment of prioritisation documents so that ‘no child with treatable cancer should die of disease in the pandemic’. Although beyond scope for this study, further work to systemically evaluate the steps and interventions that prevented delays in diagnosis during the pandemic would be a valuable academic endeavour, to draw lessons that could be applied should future similar situations arise.

We did not identify significant differences in complication rates and clinical outcomes between CYP diagnosed before and during the pandemic. Taking all complications together, CYP diagnosed during the pandemic had approximately 25% higher complication rates than those diagnosed before the pandemic, but this difference was not statistically significant.

Urgent CSF diversion may be required at initial presentation, especially in posterior fossa tumours due to tumour-related mass effect and obstructive hydrocephalus [29, 30]. Rates of requirements for urgent CSF diversion were not significantly different between groups. Rates of return to theatre within 30 days were also similar. Clinical outcomes at discharge from hospital were similar for CYP diagnosed before and during the pandemic. Service delivery changes due to COVID-19-related adaptations did not seem to cause significant disruption to participating NHS paediatric neuro-oncology services.

Although our results show a non-significant trend towards worse outcomes/higher complication rate during the pandemic, which perhaps reflects the unparalleled pressures that the health service faced, overall, they demonstrate resilience of brain tumour services. The CCLG responded promptly during the pandemic issuing guidance to NHS Trusts with practical treatment contingency plans to mitigate stretched resources again illustrating the benefit of the centralised nature of neurosurgical and neuro-oncological treatment in England.

### Limitations

This study has limitations. Incidence data from NDRS was only available for the entire nation and could not describe whether different regions were affected differently. It would be important to study regional differences with different COVID-19 restrictions during the pandemic. Additionally, cases could be missed or miscoded as data is based on individual hospitals reporting cases.

Data from three paediatric neurosurgical centres were collected retrospectively based on hospital records and are therefore prone to missing data and accuracy errors. To mitigate for this, data were extracted by clinicians with expertise in paediatric brain tumours and neurosurgical pathology. The sample size was small, especially when investigating certain complications or outcomes, and in subgroup analysis. This limits the ability to detect clinically meaningful differences, where a larger sample size with adequate statistical power could demonstrate more robust differences between the cohorts. Therefore, the results of this study should be interpreted within the study limitations, and replicating the study with a larger population would be useful to draw definitive conclusions. Nevertheless, paediatric brain tumours are not a common pathology, and in this context, studies with smaller sample size are still informative. Furthermore, severity of symptoms was not available and therefore we cannot conclude if there was a change in disease stage at presentation. Finally, important patient-level factors such as socioeconomic status, comorbidities, and ethnicity were not available within the study dataset; therefore, their impact on the outcomes of interest could not be investigated. Further work focussing on these factors would be useful to investigate how these factors affect disparities in access to care and outcomes.

Nevertheless, the results contribute to the literature demonstrating NHS acute neuro-oncology services were resilient to COVID-19-related health system changes, with comparable management and outcomes of CYP diagnosed with brain tumours during the pandemic.

## Conclusion

This is the first UK-based study investigating the effects of the COVID-19 pandemic on CYP with a new brain tumour diagnosis. Those diagnosed during the pandemic had overall similar times to diagnosis, treatment, number of symptoms, complication rates, and outcomes. Despite pandemic-related restrictions and service adaptations, acute neuro-oncology services demonstrated a degree of resilience to these changes. These findings should be validated in larger cohorts, to confirm their robustness and fully ascertain the role of specific service adaptations to paediatric neuro-oncology services during the pandemic and their true impact on patients. Finally, studies investigating the longer-term impacts of the pandemic on CYP with brain tumours are needed to provide a more holistic view of treatment-related and wider psychological consequences for patients and their families.

## Supplementary Information

Below is the link to the electronic supplementary material.

Supplementary file1 (DOCX 41 KB)

## Data Availability

No datasets were generated or analysed during the current study.
