# Supplementary material for: Impact of the COVID-19 pandemic on diagnosis and management of new brain tumours in children and young people (aged < 16 years old) in the UK
Source: Childs Nerv Syst. 2025 Aug 30;41(1):269. doi: 10.1007/s00381-025-06928-9 (PMC12398474; doi:10.1007/s00381-025-06928-9)
Supplement: Supplementary file 1 — Supplementary file1 (DOCX 41 KB) [file 381_2025_6928_MOESM1_ESM.docx]

**Supplementary material – details of subgroup analysis results**

Signs and symptoms at diagnosis by age group

*Signs and symptoms at diagnosis for children aged less than 5 years before and during the pandemic.*

| Signs and symptoms | Diagnosis **before** the pandemic, n=23 (%) | Diagnosis **during** the pandemic, n=22 (%) | Fisher’s exact p value |
| --- | --- | --- | --- |
| Headache | 6 (26.1%) | 3 (13.6%) | 0.367 |
| Nausea and/or vomiting | 10 (43.5%) | 9 (40.9%) | 1.000 |
| Motor system abnormalities | 10 (43.5%) | 7 (31.8%) | 0447 |
| Cranial nerve palsy (including ophthalmoplegia) | 4 (17.4%) | 2 (9.1%) | 0.536 |
| Visual system abnormalities | 2 (8.7%) | 1 (4.5%) | 1.000 |
| Seizures | 2 (8.7%) | 6 (27.3%) | 0.184 |
| Endocrine or growth abnormalities | 0 | 0 |  |
| Behavioural change (including lethargy) | 3 (13.0%) | 4 (18.1%) | 1.000 |
| Other (yes / no) | 5 (21.7%) | 6 (27.3%) | 1.000 |
| Number of symptoms at presentation | 1 symptom = 10  2 symptoms = 4  3 symptoms = 8 | 1 symptom = 9  2 symptoms = 10  3 symptoms = 3 | 0.100 |

*Signs and symptoms at diagnosis for children aged 5-11 years before and during the pandemic.*

| Signs and symptoms | Diagnosis **before** the pandemic, n=38 (%) | Diagnosis **during** the pandemic, n=27 (%) | Fisher’s exact p value |
| --- | --- | --- | --- |
| Headache | 27 (71.5%) | 17 (63.0%%) | 0.574 |
| Nausea and/or vomiting | 17 (44.7%) | 11 (40.7%) | 0.883 |
| Motor system abnormalities | 7 (18.4%) | 5 (18.5%) | 1.000 |
| Cranial nerve palsy (including ophthalmoplegia) | 4 (10.5%) | 5 (18.5%) | 0.693 |
| Visual system abnormalities | 13 (34.2%) | 9 (33.3%) | 1.000 |
| Seizures | 2 (5.3%) | 4 (14.8%) | 0.296 |
| Endocrine or growth abnormalities | 3 (7.9%) | 4 (14.8%) | 1.000 |
| Behavioural change (including lethargy) | 1 (4.0%) | 3 (21.4)%) | 0.674 |
| Other (yes / no) | 2 (5.3%) | 5 (18.5%) | 0.165 |
| Number of symptoms at presentation | 1 symptom = 12  2 symptoms = 13  3 symptoms = 10  4 symptoms = 2  5 symptoms = 0 | 1 symptom = 9  2 symptoms = 7  3 symptoms = 7  4 symptoms = 3  5 symptoms = 1 | 0.704 |

*Signs and symptoms at diagnosis for children aged 12-16 years before and during the pandemic.*

| Signs and symptoms | Diagnosis **before** the pandemic, n=27 (%) | Diagnosis **during** the pandemic, n=22(%) | Fisher’s exact p value |
| --- | --- | --- | --- |
| Headache | 13 (48.1%) | 16 (72.7%) | 0.143 |
| Nausea and/or vomiting | 2 (7.4%) | 10 (45.5%) | **0.002*** |
| Motor system abnormalities | 2 (7.4%) | 8 (36.4%) | **0.029*** |
| Cranial nerve palsy (including ophthalmoplegia) | 2 (7.4%) | 1 (4.5%) | 1.000 |
| Visual system abnormalities | 9 (33.3%) | 5 (22.7%) | 0.530 |
| Seizures | 5 (18.53%) | 5 (22.7%) | 0.737 |
| Endocrine or growth abnormalities | 2 (7.4%) | 2 (9.0%) | 1.000 |
| Behavioural change (including lethargy) | 1 (3.7%) | 1 (4.5%) | 1.000 |
| Other (yes / no) | 4 (14.8%) | 1 (1.5%) | 0.362 |
| Number of symptoms at presentation | No symptoms = 5  1 symptom = 9  2 symptoms = 9  3 symptoms = 3  4 symptoms = 1 | No symptoms = 0  1 symptom = 6  2 symptoms = 7  3 symptoms = 7  4 symptoms = 2 | 0.112 |

Complications by age group

*Complications and outcomes for children aged less than 5 years before and during the pandemic.*

| Complications and clinical outcomes | Diagnosis **before** the pandemic (%) | Diagnosis **during** the pandemic (%) | Fisher’s Exact p-value |
| --- | --- | --- | --- |
| Requirement for urgent CSF diversion | 7 (30.4%) | 8 (36.4%) | 0.758 |
| Complications (any) | 10 (43.5%) | 9 (40.9%) | 1.000 |
| Return to theatre within 30 days | 5 (21.7%) | 5 (22.7%) | 1.000 |
| **Clinical outcomes** | Well = 15  Residual deficit = 6  New deficit = 2  Died = 0 | Well = 9  Residual deficit = 9  New deficit = 3  Died = 1 | 0.360 |
| Poor outcome (New deficit or died) | 2 (8.7%) | 4 (18.2%) | 0. 414 |

*Complications and outcomes for children aged 5-11 years before and during the pandemic.*

| Complications and clinical outcomes | Diagnosis **before** the pandemic (%) | Diagnosis **during** the pandemic (%) | Fisher’s Exact p-value |
| --- | --- | --- | --- |
| Requirement for urgent CSF diversion | 8 (21.1%) | 2 (7.4%) | 0.175 |
| Complications (any) | 9 (23.7%) | 12 (44.4%) | 0.108 |
| Return to theatre within 30 days | 7 (18.4%) | 7 (25.9%) | 0.547 |
| **Clinical outcomes** | Well = 27  Residual deficit = 5  New deficit = 5  Died = 1 | Well = 18  Residual deficit = 3  New deficit = 5  Died = 1 | 0.926 |
| Poor outcome (New deficit or died) | 6 (15.8%) | 6 (22.2%) | 0.534 |

*Complications and outcomes for children aged 12 years and over before and during the pandemic.*

| Complications and clinical outcomes | Diagnosis **before** the pandemic (%) | Diagnosis **during** the pandemic (%) | Fisher’s Exact p-value |
| --- | --- | --- | --- |
| Requirement for urgent CSF diversion | 5 (18.5%) | 4 (18.2%) | 1.000 |
| Complications (any) | 6 (22.2%) | 8 (36.4%) | 0.348 |
| Return to theatre within 30 days | 0 | 3 (13.6%) | 0.084 |
| **Clinical outcomes** | Well = 20  Residual deficit = 3  New deficit = 4  Died = 0 | Well = 15  Residual deficit = 1  New deficit = 5  Died = 1 | 0.589 |
| Poor outcome (New deficit or died) | 4 (14.8%) | 6 (27.3%) | 0.311 |

Complications by tumour type

*Complications and clinical outcomes for each of the three most common tumour types: pilocytic astrocytoma, ependymoma, medulloblastoma.*

|  | Diagnosis **before** the pandemic (%) | Diagnosis **during** the pandemic (%) | Fisher’s Exact p-value |
| --- | --- | --- | --- |
| **Pilocytic astrocytoma** | | | |
| Complications (any) | 4 (26.7%) | 7 (36.8%) | 0.715 |
| Return to theatre within 30 days | 1 (6.7%) | 2 (10.5%) | 1.000 |
| Discharge outcome | Good: 13  Poor: 2 | Good: 14  Poor: 5 | 0.209 |
| **Ependymoma** | | | |
| Complications (any) | 3 (42.9%) | 4 (80%) | 0.293 |
| Return to theatre within 30 days | 2 (28.5%) | 4 (80%) | 0.242 |
| Discharge outcome | Good: 7  Poor: 0 | Good: 3  Poor: 2 | 0.152 |
| **Medulloblastoma** | | | |
| Complications (any) | 8 (53.3%) | 7 (77.8%) | 0.356 |
| Return to theatre within 30 days | 3 (25.0%) | 4 (44.4%) | 0.357 |
| Discharge outcome | Good: 11  Poor: 4 | Good: 5  Poor: 4 | 0.571 |
| **Low grade glioma** | | | |
| Complications (any) | 2 (25.0%) | 2 (33.3%) | 1.000 |
| Return to theatre within 30 days | 0 | 1 (26.7%) | 0.427 |
| Discharge outcome | Good: 7  Poor: 1 | Good: 5  Poor: 1 | 1.000 |
